# The efficacy and safety of fire needle for the treatment of lumbar disc herniation: A protocol for systematic review and meta-analysis

**DOI:** 10.1097/MD.0000000000023751

**Published:** 2020-12-11

**Authors:** Meiyuan Wang, Beisi Zheng, Cunshu Wu, Shixiong Yi

**Affiliations:** a Chongqing Traditional Chinese Medicine Hospital Rehabilitation Department, Chongqing; b Clinical Medical College of Acupuncture, Moxibustion and Rehabilitation, Guangzhou University of Chinese Medicine, Guangzhou, China.

**Keywords:** fire needle, lumbar disc herniation, network meta-analysis, protocol, systematic review

## Abstract

**Background:**

A very large acceleration in clinical studies on the efficacy of fire needle to treat lumbar disc herniation (LDH) are increasing, while studies on the assessment of its efficacy are still lacking. Therefore, this study will demonstrate the efficacy of fire needle in the treatment of LDH combining with the meta-analysis.

**Methods:**

The studies on randomized controlled trials (RCTs) will be searched at the databases of China National Knowledge Infrastructure (CNKI), WANFANG database (Chinese Medicine Premier), Chinese Scientific Journal Database (VIP), Chinese Biomedical Literature database (CBM), PubMed, EMBASE, and Cochrane Library from their inception to May 1, 2020.

**Results:**

This authentic and multi-dimensional study will shed light on the referable information for the treatment of LDH with fire needle.

**Conclusion:**

This study will evaluate the efficacy of fire needle in the treatment of LDH.

**Registration:**

PROSPERO (registration number CRD42020158596).

## 1 Introduction

Lumbar disc herniation (LDH) refers to a displacement of disc material (nucleus pulposus or annulus fibrosis) beyond the inter-vertebral disc space, which may result in back pain or radicular symptoms of the lower extremities due to degeneration disc and exterior pressure on the spinal cord or nerve root.^[1]^ The incidence of lumbar disc herniations is high, it affects nearly 2% to 3% of the world's population, while the incidence of LDH in China is approximately 7.62%.^[2]^ LDH has brought economic and psychologic burdens to many families and a heavy burden on social care. It mainly occurs among working adults aged <50 years and has become an occupational health issue. As a common and frequently occurring disease, LDH leads to a reduction in quality of people's daily life, work, and lifestyle.^[3,4]^ What's more, the treatment of LDH brought high costs. The economic financial burden of treating LDH in the United States is more than a billion dollars, with nearly $300 million dedicated to surgical procedures.^[5]^

At present, the clinical treatment of LDH is mainly performed by surgical and conservative treatments.^[6]^ It is reported that only 15% to 20% of LDH patients with LDH clinically required surgery because of severe neurological symptoms.^[7]^ Currently, the efficacy of surgical procedure relative to nonoperative care remains controversial.^[8]^ Due to the high recurrent rate of LDH, most clinic doctors advise patients to accept nonoperative treatments.^[9]^ What's more, surgical treatments may also results in the paravertebral muscles, nerve retraction, and lumbar instability after surgery.^[10,11]^ Therefore, conservative treatments are widely used for LDH, as it has minor side effects and is more economical.^[12]^

Fire needle is an ancient acupuncture with the original record in Huangdi Neijing. Compared with filiform needle, fire needle could bolster acupuncture and warm effect. The acupoints of fire needle therapy will be the same as those in the filiform needle except for needling depth, acupuncture manipulation, and needle retention time.^[13]^ Recent studies have shown that fire needle can improve clinical symptoms and relieve pain of LDH.^[14]^ Fire needle has the functions of enhancing immunity, regulating blood circulation, and preventing diseases.^[15]^ From the perspective of modern medicine, the heat provided by fire needles promotes microcirculation in the lesion area through the regulation of cutaneous nerves, which is beneficial for the absorption of inflammation and metabolites. Furthermore, the high temperature of fire needles directly kills the microorganisms in the nodules and achieves anti-inflammatory effects.^[16]^

Nowadays, a fresh wave of positivity pulsates emerges on the treatment of LDH with acupuncture. Nevertheless, the studies of fire needle and LDH are scattered without targeted systematic reviews to confirm the efficacy of fire needle. Hence, the purpose of this study is to determine through a meta-analysis whether fire needle has better results for LDH.

## 2 Methods

### 2.1 Study registration

The protocol of this systematic review and meta-analysis has been registered on PROSPERO (CRD42020158596). (https://www.crd.york.ac.uk/prospero/). We will conduct this protocol with being in line with the guidelines of Preferred Reporting Items for Systematic Reviews and Meta-Analyses protocols (PRISMA-P).^[17]^

### 2.2 Search strategy

The adopting databases will include China National Knowledge Infrastructure (CNKI), WANFANG database (Chinese Medicine Premier), Chinese Scientific Journal Database (VIP), Chinese Biomedical Literature database (CBM), PubMed, EMBASE, and Cochrane Library. We will search studies in Chinese and English between inception and May 1, 2020. Search terms of MeSH and free text terms will be used to identify relevant studies. The specific details of search strategy in PubMed will be demonstrated in Table 1.

**Table 1:** Search strategy in PubMed.

Number	Search items
#1	“fire needle” [MeSH Terms] OR “fire acupuncture” [Title/Abstract] OR “huozhen” [Title/Abstract] OR “quench needle” [Title/Abstract] OR “red-hot needle” [Title/Abstract]
#2	“Lumbar disc herniation” [MeSH Terms] OR “Lumbar Intervertebral Disc Herniation” [Title/Abstract] OR “Lumbar Disc Protrusion” [Title/Abstract] OR “Degenerative Intervertebral Discs” [Title/Abstract] OR “Prolapse of Lumbar Intervertebral Disc” [Title/Abstract]
#3	“randomized controlled trial” [Publication Type]
#4	#1 AND #2 AND #3

### 2.3 Eligibility criteria

#### 2.3.1 Type of study

With the aim of shedding light on the beneficial effects of the treatments, we will review the randomized controlled trials (RCT) of fire needle for LDH. The studies will be restricted for the language of Chinese and English, but not for the blinding and allocation concealment.

#### 2.3.2 Participants

All patients were diagnosed with LDH through any recognized diagnostic criteria, whose age, sex, education, region, race, and course of LDH.

#### 2.3.3 Interventions

The experimental group must be merely treated with fire needle, while the control group accepted other therapies except fire needle.

#### 2.3.4 Outcomes

The primary outcome measures targeted in our study are efficacy, pain (e.g., Japanese Orthopaedic Association [JOA], visual analogue scales [VAS], and present pain intensity [PPI]), and function (e.g., Oswestry disability index [ODI]). The secondary outcome measures are the quality of life (e.g., 36-item Short-Form Health Survey [SF-36]), and side-effects.

### 2.4 Selection of studies and extraction of data

#### 2.4.1 Study selection

**Figure 1:**
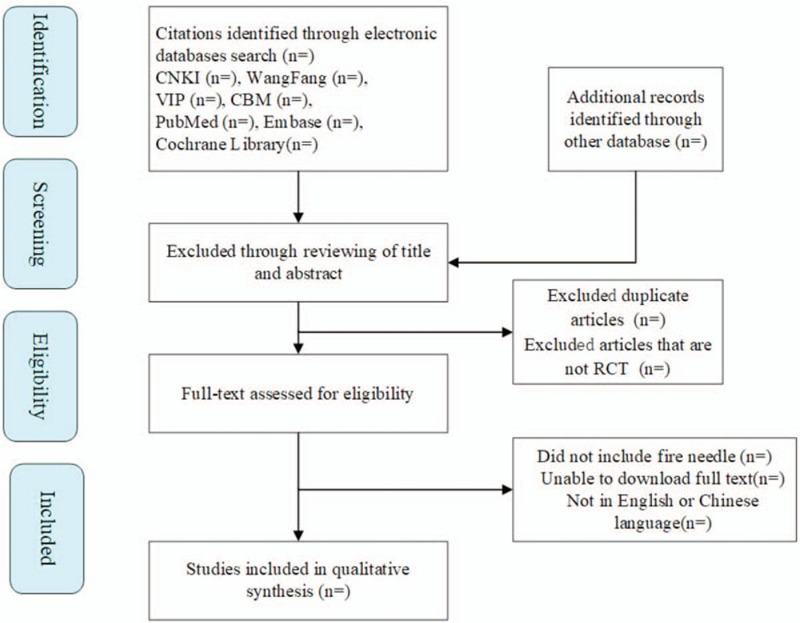
Flow diagram of studies identified.

Two reviewers will independently conduct screening process and data extraction. We will negotiate with a third reviewer to cope with discrepancy if the disagreement emerges. In the literature screening, we will use EndNote X7 with titles, abstracts, and full-text of studies, bringing convenience to determine the included studies. The selection process is demonstrated in Fig. 1.

#### 2.4.2 Data extraction

The authors will independently extract data following the standardized sheet recommended by the Cochrane Handbook of Systematic Reviews of Interventions. The extracted information recorded in Excel 2010 includes title, first author, year of publication, country or region, study design, sample size, participants, intervention, outcomes, and adverse events.

### 2.5 Risk of bias assessment

The authors will adopt the Cochrane Collaboration recommendations to assess risk of bias,^[18]^ conducting 7 items of assessment: random sequence generation, allocation concealment, blinding of participants and personnel, blinding of outcome assessments, incomplete outcome data, selective reporting, and other sources of bias. Every of them can be divided into Low risk of bias, Unclear risk of bias, and High risk of bias. We will negotiate with a third reviewer when the disagreement emerges.

### 2.6 Statistical analysis

We will conduct statistical analysis by Revman 5.3 provided by the Cochrane Collaboration and STATA 15.0. Heterogeneity among the studies was evaluated using the chi-square test and *I*^2^ statistic. The option of analytical model will depend on the value of *I*^2^. If *P* > .01, *I*^*2*^ < 50%, demonstrating there is heterogeneity, we are supposed to conduct a meta-analysis by a fixed-effect model. And, if *P* < .10, *I*^2^ > 50%, we will choose a random-effect model. In the analysis of dichotomous outcomes, we will analyze through relative ratio (RR), while we will calculate weighted mean difference (WMD), standardized mean difference (SMD) in continuous outcomes. We will report 95% confidence intervals (CIs) for all outcomes.

### 2.7 Subgroup analysis

Subgroup analysis will be performed depending on control intervention and different outcomes.

### 2.8 Sensitivity analysis

We will remove included studies one by one and record the changes in the combined effects to obverse the change of heterogeneity. When the heterogeneity changes after excluding a study, the origin of heterogeneity may be this study. Then, we could analyze the heterogeneity from study design, sample size, outcomes, etc.

### 2.9 Assessment of reporting biases

We will conduct assessment reporting biases by funnel plot to observe the asymmetry of funnel plot.

### 2.10 Quality of evidence

The quality of evidence will be assessed on the Grading of Recommendations Assessment, Development, and Evaluation (GRADE) working group approach, which divides the scientific evidence into high, moderate, low, and very low.^[19]^

### 2.11 Ethical approval

Ethical approval is not necessary in this protocol for no patients being involved.

## 3 Discussion

Due to the lack of quantitative analysis of fire needle treatment for LDH, clinicians could not definitely master the efficacy of fire needle in the treatment of LDH. Therefore, meta-analysis will be used to demonstrate the efficacy and safety of fire needle in the treatment of LDH in some extent. Relevant people will show a great affection for our results, including orthopedists, acupuncturists, researcher LDH patients, etc. However, with the rapid development of research, some new studies could not be included. Moreover, the limited inclusion of Chinese and English studies may affect the selection bias. In sum, our study could fills in the gaps of quantitative analysis and usher in more benefits in fire needle and LDH within the bounds of expectations.

## Author contributions

**Conceptualization:** Meiyuan Wang.

**Data curation:** Beisi Zheng.

**Methodology:** Beisi Zheng.

**Validation:** Shixiong Yi.

**Writing – original draft:** Beisi Zheng, Cunshu Wu.

**Writing – review & editing:** Meiyuan Wang.
